# Histone methyltransferase MET-2 differentially regulates autosome and X-chromosome structure and transcription during meiosis in *C. elegans*

**DOI:** 10.64898/2026.07.26.740854

**Published:** 2026-07-29

**Authors:** Carolyn Remsburg, Aimee Jaramillo-Lambert

## Abstract

During meiosis, accurate chromosome segregation requires significant condensation and compaction. These processes are mediated by condensins, cohesins, and histone tail modifications. We identified that MET-2, a histone methyltransferase that catalyzes the dimethylation of histone H3 lysine 9 (H3K9me2), differentially impacts chromosome size in the male vs. female *C. elegans* germline. In *met-2* null worms, autosomes during spermatogenesis are significantly larger than wild type, while chromosome size during oogenesis is unaffected. X-univalent size in males is also unaffected by loss of MET-2, indicating MET-2 differentially regulates autosomal and X-chromosome compaction in male spermatogenesis. Autosome size is not changed when males harbor a catalytically deficient MET-2 *(met-2* CD) or have mutations preventing germline histone H3K9 methylation (H3K9R). In addition, *met-2* males, in contrast to *met-2* CD or H3K9R males, have more active RNA pol II in later stages of meiosis. These data suggest MET-2 plays a noncatalytic role in mediating chromosome structure and transcription. In *met-2* male germ lines, genes on the X chromosome, which is typically enriched in H3K9me2, are significantly more likely to be upregulated than genes on autosomes, even though X-univalent size is unchanged. These results suggest that MET-2 plays a sex-specific role that is not limited to its enzymatic activity.

## Full Text Availability

The license terms selected by the author(s) for this preprint version do not permit archiving in PMC. The full text is available from the preprint server.

